# Bar-cas12a, a novel and rapid method for plant species authentication in case of *Phyllanthus amarus* Schumach. & Thonn

**DOI:** 10.1038/s41598-021-00006-1

**Published:** 2021-10-22

**Authors:** Kittisak Buddhachat, Suphaporn Paenkaew, Nattaporn Sripairoj, Yash Munnalal Gupta, Waranee Pradit, Siriwadee Chomdej

**Affiliations:** 1 Department of Biology, Faculty of Science, Naresuan University, Phitsanulok, 65000 Thailand; 2 Department of Agricultural Science, Faculty of Agriculture, Natural Resources and Environment, Center of Excellence in Research for Agricultural Biotechnology, Naresuan University, Phitsanulok, 65000 Thailand; 3 Department of Biology, Faculty of Science, Research Center in Bioresources for Agriculture, Industry and Medicine, Chiang Mai University, Chiang Mai, 50200 Thailand

**Keywords:** Analytical biochemistry, Plant sciences, Plant biotechnology

## Abstract

Rapid and accurate species diagnosis accelerates performance in numerous biological fields and associated areas. However, morphology-based species taxonomy/identification might hinder study and lead to ambiguous results. DNA barcodes (Bar) has been employed extensively for plant species identification. Recently, CRISPR-cas system can be applied for diagnostic tool to detect pathogen’s DNA based on the collateral activity of cas12a or cas13. Here, we developed barcode-coupled with cas12a assay, “Bar-cas12a” for species authentication using *Phyllanthus amarus* as a model. The gRNAs were designed from *trnL* region, namely gRNA-A and gRNA-B. As a result, gRNA-A was highly specific to *P. amarus* amplified by RPA in contrast to gRNA-B even in contaminated condition. Apart from the large variation of gRNA-A binding in DNA target, cas12a- specific PAM’s gRNA-A as TTTN can be found only in *P. amarus*. PAM site may be recognized one of the potential regions for increasing specificity to authenticate species. In addition, the sensitivity of Bar-cas12a using both gRNAs gave the same detection limit at 0.8 fg and it was 1,000 times more sensitive compared to agarose gel electrophoresis. This approach displayed the accuracy degree of 90% for species authentication. Overall, Bar-cas12a using *trnL*-designed gRNA offer a highly specific, sensitive, speed, and simple approach for plant species authentication. Therefore, the current method serves as a promising tool for species determination which is likely to be implemented for onsite testing.

## Introduction

Species authentication/discrimination is an essential task in various areas in biology systematics, ecology, evolution, forensics, food science, medical as well as even herbal and cosmetic industries, leading to correct species exploitation regarding their purposes^1–8^. Traditional species taxonomy has been performed using the external morphological features or microanatomy which tightly requires the complete flower features or complete significant characteristics for species identification by an expert^9^. In some instances, the obtained samples have been incomplete forms, immature stage, or modified/processed samples without key characters to identify, making difficulty or almost impossible to identify species and impeding the progress of investigation or research^9^. In several decades ago, advanced molecular approaches e.g., hybridization, DNA fingerprint, DNA barcodes, high resolution melting (HRM) have been used widely and extensively for facilitating species authentication in various organisms^2–6,10–16^. Certainly, these molecular approaches enable species identification despite the specimens with completely damaged but DNA existing, especially DNA barcodes (Bar) which there are many regions exhibiting a successful species discrimination for plant species (e.g., *rbcL, matK*, *trnL*, and ITS)^15,16^. However, they are relatively complex, time-consuming, and expensive because they necessitate the use of costly equipment (e.g., thermal cycler, realtime PCR, sequencer machine).

Currently, nucleic acid isothermal amplification (e.g., RCA, LAMP and RPA) has been emerging and gaining attention for RNA/DNA amplification, in particular pathogen detection as they require only heat box or water bath, leading to adaptation for point-of-care testing^17–19^. RPA is one of isothermal amplification based on enzymatic activities relating to DNA replication process and the reaction can be performed at constant temperature in range of 30–45 °C for DNA amplification (optimal temperature at 37 °C)^18,19^, mycoplasma^20^, and virus/viroid RNA in plant^21^.

Recently, CRISPR-cas systems exhibited the high potential for genome editing with accuracy and precise in the specific DNA target and included the adaptation for pathogen diagnostic with high sensitivity, specificity, simplicity, and speed, for instances, HPV-16 and 18^22,23^ and shrimp pathogens e.g., white spot syndrome virus (WSSV)^24^. The cas12a can be applied as diagnostic tool because it has the collateral activity or trans-activity for cleavage of non-target single stranded (ss) DNA once forming a tertiary complex (cas12a-gRNA-target)^25^. ssDNA is designed as reporter based on fluorescence resonance energy transfer (FRET) between fluorescence and its quencher or antigen–antibody interaction by lateral flow dipstick readout^22–24,26^.

Herein, we would like to establish a novel method for plant species authentication with the combination of plant DNA barcode, *trnL* and cas12a, namely “Bar-cas12a”. In this study, *Phyllanthus* species including *Phyllanthus amarus*, *Phyllanthus urinaria*, *Phyllanthus debilis*, *Phyllanthus virgatus*, were used as a model to validate Bar-cas12a for species authentication of *P. amarus* because they have similar morphological features and have been used as herbal commercialized products.

## Results

### Condition optimization of cas12a assay

**Figure 1:**
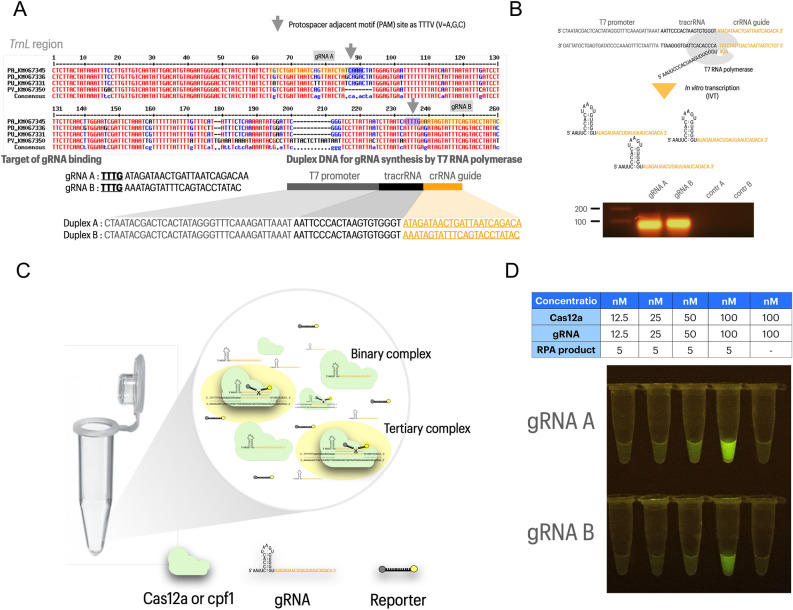
The preparation of cas12a condition for species authentication. (**A**) gRNA-A and gRNA-B design based on the multiple alignment of *trnL* loci and the construction of double stranded DNA as temples for gRNA synthesis. (**B**) The synthesis of gRNA-A and gRNA-B by in vitro transcription with T7 RNA polymerase and the synthesized gRNAs were detected by agarose gel electrophoresis. (**C**) The schematic illustrates the mechanism of cas12a to form binary and tertiary complex to cleave the reporter single stranded DNA as result of the fluorescence signal. (**D**) The condition optimization of in vitro digestion of cas12a by varying the concentration ratio of cas12a and gRNA at 1: 1 to produce the fluorescence at 37 °C for an hour. *Phyllanthus* species were studied including *Phyllanthus amarus* (PA), *Phyllanthus urinaria* (PU), *Phyllanthus debilis* (PD), *Phyllanthus virgatus* (PV). Initial image of agarose gel electrophoresis is shown in Figure S1.

In this study, we presented two gRNAs designed from *trnL* region which were specific to *P. amarus*. The gRNA-A were designed in the opposite direction to gRNA-B and the variation nucleotide in binding site of gRNA-A was more diverse than that of gRNA-B which existed only single mismatch (Fig. 1A). Both of two gRNAs for cas12a assay were successfully produced by in vitro transcript which duplex DNAs for two gRNAs were used as the templates for gRNA synthesis as depicted in Fig. 1A,B. The scheme illustration for the principle of in vitro digestion of cas12a was shown in Fig. 1C. In addition, the concentration of cas12a and gRNA gave the highest fluorescence from the cleavage of ssDNA reporter at 37 °C for an hour was at 100 nM: 100 nM whereas there was no fluorescence in control (without DNA target) (Fig. 1D).

### Species authentication performance of Bar-cas12a

**Figure 2:**
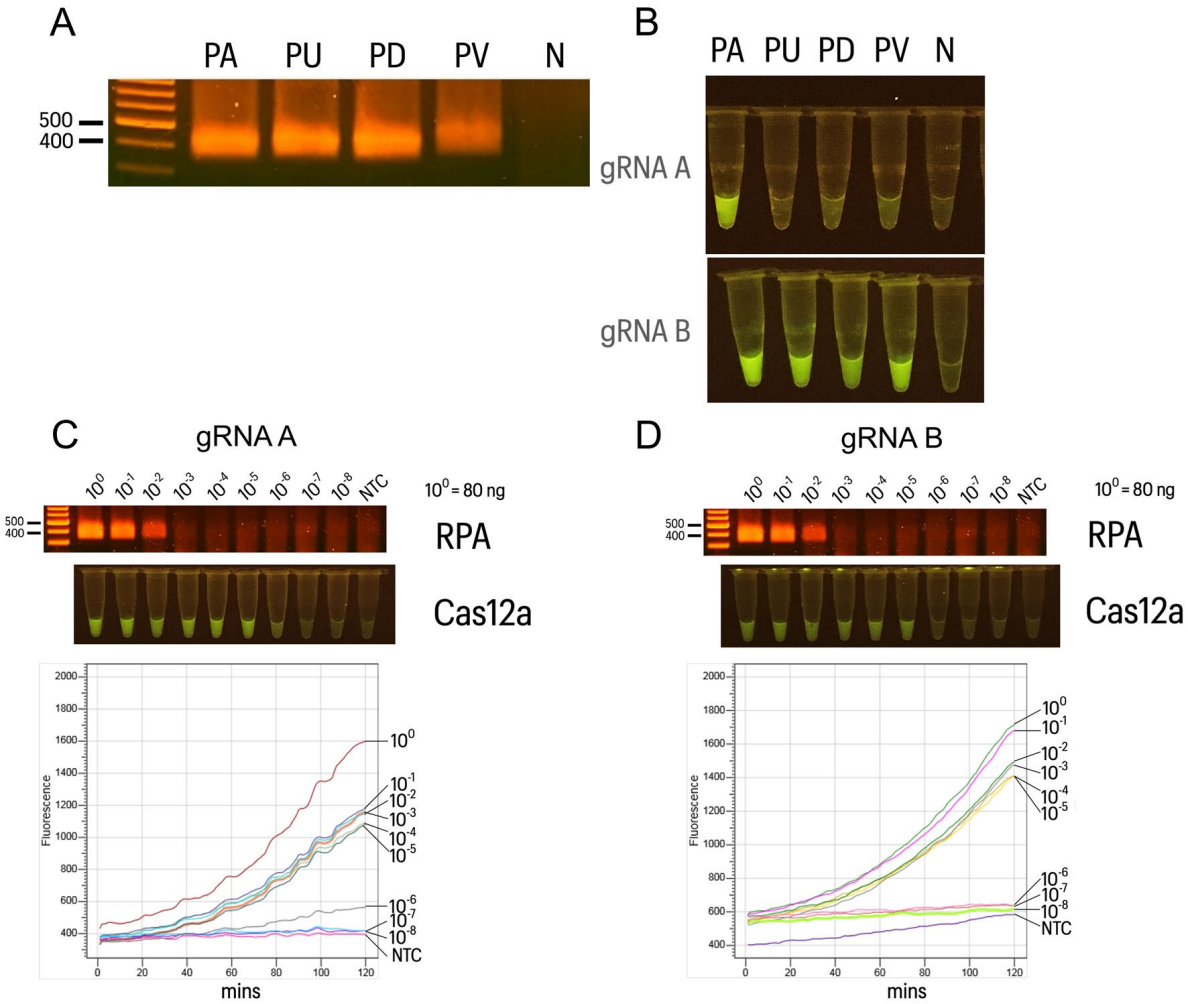
Specificity and sensitivity of Bar-cas12a for *P. amarus* authentication. (**A**) DNA amplification for the four species including *P. amarus* (PA), *P. urinaria* (PU), *P. debilis* (PD) and *P. virgatus* (PV) by RPA using universal *trnL* primer. (**B**) Specificity test by Bar-cas12a using gRNA-A and gRNA-B. (**C**,**D**) Sensitivity test by PCR and Bar-cas12a using gRNA-A and gRNA-B and the fluorescence signal were monitored for cleavage of ssDNA reporters by realtime PCR within two hours. Initial images of agarose gel electrophoresis are shown in Figure S2 and S3, respectively.

To evaluate the performance of Bar-cas12a assay for species authentication of *P. amarus*. For specificity determination, *trnL* region of different four *Phyllanthus* species were amplified by RPA using modified universal *trnL* primer, producing approximately 400 bp DNA fragment (Fig. 2A) and RPA products of four species were used for cas12a using gRNA-A and gRNA-B. Our results displayed that gRNA-A gave the fluorescence signal with specific PA while gRNA-B was positive fluorescence signal for all species tested (Fig. 2B). In addition, the sensitivity assay was done using the different starting amount of *P. amarus* DNA for amplification by RPA in range of 0–80 ng. We discovered that the limit of detection (LOD) was 0.8 ng for RPA amplicons under agarose gel electrophoresis which yielded a DNA band as shown in Fig. 2C,D. Meanwhile, positive fluorescence signals for Bar-cas12a using gRNA-A and gRNA-B were observed in the range of 0.8 pg–80 ng for both gRNAs, exhibiting substantially higher sensitivity than the RPA amplicon visualization by agarose gel electrophoresis (Fig. 2C,D).

**Figure 3:**
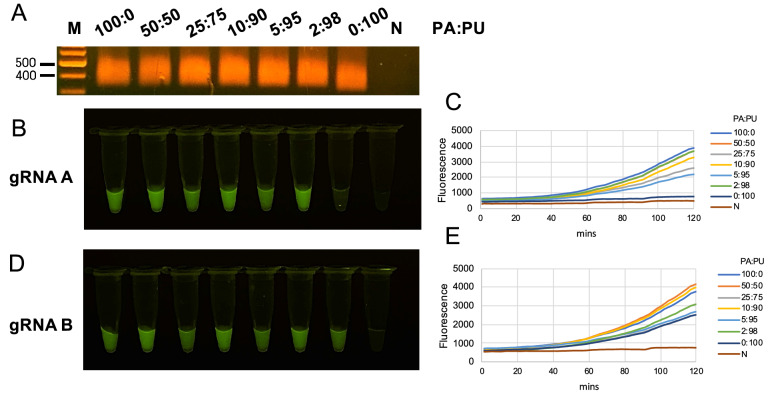
Species authentication by Bar-cas12a in admixture samples. (**A**) DNA amplification of DNA admixture in different proportions by RPA using *trnL* loci. DNA admixture between *P. amarus* (PA) and *P. urinaria* (PU) were done in different amount proportions as 100%, 50%, 25%, 10%, 5%, 2% and 0% of PA contaminated with PU which stock concentration of each species used was 10 ng/µl. (**B**,**D**) In vitro digestion of cas12a using gRNA A and gRNA B and observed under LED transilluminator. (**C**,**E**) the acquisition of fluorescence signal by realtime PCR over two hours. Initial images of agarose gel electrophoresis are shown in Figure S4.

In addition, species authentication by Bar-cas12a was validated using the admixture between *P. amarus* and *P. urinaria* with different amount of DNA proportion. Indeed, all admixtures gave the positive for DNA amplification by RPA (Fig. 3A). However, Bar-cas12a with gRNA-A demonstrated greater capability of species authentication with high specificity than gRNA-B because Bar-cas12a with gRNA-B produced positive results (100%) for *P. urinaria* but not with gRNA-A (Fig. 3B–E). Moreover, we found that Bar-cas12a using gRNA-A produced positive results even with a relatively small amount of *P. amarus* DNA (only 2%) in the admixture condition (Fig. 3B,C).

**Figure 4:**
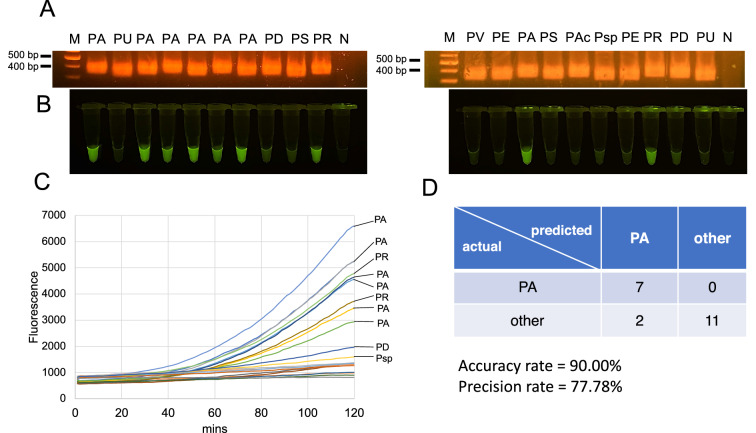
Accuracy test for *P. amarus* determination by Bar-cas12a using *trnL*. (**A**) DNA amplification by RPA on agarose gel of the different *Phyllanthus* species including *P. amarus* (PA), *P. urinaria* (PU), *P. debilis* (PD), *P. virgatus* (PV)*, P. airy-shawii* (PS)*, P. acidus* (PAc)*, P. emblica* (PE)*, P. reticulatus* (PR), *Phyllanthus* sp. (Psp). Cas12a assay for detecting PA-specific RPA product by naked eye under LED transilluminator (**B**) and fluorescence signal by a realtime PCR machine (**C**). (**D**) The confusion matrix of actual and predicted species with accuracy rate and precision rate of *P. amarus* authentication. Initial images of agarose gel electrophoresis are shown in Figure S5.

Herein, we attempted to validate the efficacy of *P. amarus* identification by Bar-cas12a using *trnL* among the different species of *Phyllanthus*. In addition, the assay was performed to non-herbaceous species, the person performing this assay was blinded to the actual species. Although DNA amplification by RPA gave the positive amplicon for all samples (Fig. 4A), there were 11 samples with positive fluorescence for cas12a assay under LED transilluminator (Fig. 4B) and a realtime PCR machine (Fig. 4C). This method exhibited a great performance to determine actual *P. amarus* with 100%, but there were only two samples of *P. reticulatus* with positive results of cas12a assay. The accuracy and precision degree of the assay was 90.00% and 77.78%, respectively (Fig. 4D).

## Discussion

CRISPR-cas system has not been only accomplished for genome editing in the certain target for various organisms, but it can also be adapted for efficient diagnostic tool with high sensitivity and specificity to detect the pathogens^20–24,26^. Furthermore, this approach has a high potential for using onsite testing as a quick process that does not require sophisticated equipment^20–24,26^. This is the first report to apparently exhibit the feasibility of combining DNA barcode and cas12a assay to authenticate plant species as *P. amarus*. Our significant findings demonstrated that Bar-cas12a using gRNA-A of *trnL* barcode based on RPA enabled to specifically authenticate *P. amarus* with high sensitivity of LOD at 0.8 fg which was three orders of magnitude more sensitive than RPA visualized by agarose gel electrophoresis. Additionally, Bar-cas12a using gRNA-A enables species authentication of *P. amarus* even after contaminated with *P. urinaria* and offers high accuracy degree of 90%.

In the present study, gRNA-A and gRNA-B were designed based on the *trnL* sequences derived from the different four species including *P. amarus*, *P. urinaria*, *P. debilis* and *P. virgatus* because of the high variation sequences^27,28^. Although all of them share common morphological features, *P. amarus* is a single species existing bioactive compound of phyllanthin and hypophyllathin responsible for hepatoprotection^29^. *P. amarus* has been commercialized as various product forms e.g., tea infusion, capsule, and tablets^6^. Thus, *P. amarus* was employed as a plant model to authenticate by Bar-cas12a assay. For cas12a condition, the adequate and suitable concentration ratio of cas12a and gRNA to form binary complex and trigger the activity for positive fluorescence within an hour was 100 nM: 100 nM. Several studies have shown that the optimal concentration ratio between cas12a and gRNA for detecting targets can be as high as 200 nM: 500 nM20 and as low as 30 nM: 36 nM^22^.

The specificity and sensitivity of Bar-cas12a using either gRNA-A or gRNA-B for *P. amarus* authentication were determined. Obviously, the specificity of gRNA-A was the *P. amarus*- specific marker due to other species without the positive fluorescence whereas gRNA-B gave the positive for four herbaceous species. Furthermore, we verified the species authentication ability of Bar-cas12a in contaminated conditions with unwanted species by admixing different amounts of *P. amarus* and *P. urinaria* DNA. Our findings demonstrated that Bar-cas12a using gRNA-A is highly species-specific to *P. amarus* rather than using gRNA-B.

As of the multiple alignment of DNA target for gRNA design across four species, in gRNA-A, the suitable sequence of PAM site exists only in *P. amarus* whereas *P. debilis* and *P. urinaria*’s PAM site differ from *P. amarus* and it is absence in *P. virgatus*. Besides, the DNA target for binding to gRNA-A of *P. debilis* carries the different nucleotide from *P. amarus* at the first base of seed region (first 1–5 nt at 3’ next to PAM site) which is intolerant region to a mismatch^25^ and *P. urinaria* contains difference in three nucleotides (out of seed region) from *P. amarus*. In contrast, DNA target to bind to gRNA-B of three species carries only a single variable site although a variable site (A > G) was in second position of seed regions (1–5 first base next to PAM site)^25^ with conserved PAM site. Regarding this, gRNA-A showed better species differentiability than gRNA-B. Taken together, we concluded that there are two components that should be considered to select DNA target for gRNA design for species identification /discrimination; (1) the variation in PAM site and (2) the nucleotide difference in DNA target for gRNA binding across species. However, although Bar-cas12a using *trnL* provided a high accuracy degree of 90.00%, the precision was somehow low with ~ 78% because this method seemed to give the positive fluorescence in *P. reticulatus*, a non-herbaceous *Phyllanthus*, which may have resulted in unaddressed adulteration with *P. amarus* during the experiment. For multiple alignment of *trnL* region including *P. reticulatus* (Figure S6), it was obvious that although PAM sites for gRNA-A on *P. reticulatus* differed from *P. amarus*, the gRNA sequence is identical, possibly leading to cas12a-gRNA-A enabling to bind to *P. reticulatus*’s DNA target and trigger trans-activity, yielding a positive signal. For sensitivity assay, Bar-cas12a using both gRNA-A and gRNA-B provide the LOD at 0.8 fg which was three orders of magnitude more sensitive than agarose gel electrophoresis-visualized RPA. This indicated that the hyphenation of cas12a enable to increase the sensitivity via the signal amplification of DNase activity of cas12a triggered by specific DNA target. Cas12a coupled with nucleic acid amplification such as LAMP or RPA has been achieved for detecting plant RNA viruses^21^, HPV16 and HPV18^22,23^ and bacterial contamination in food^30^, with high specificity and sensitivity.

Here, we describe the feasibility of implementing cas12a combined with isothermal nucleic amplification of DNA barcode region by RPA to facilitate species authentication of *P. amarus*. This method allows for species authentication to be completed in two hours which consisted of DNA amplification (> 30 min) and cas12a cleavage to detect specific PCR product of *P. amarus* (> 90 min) which was performed in a tube assay dependent only heat box/water bath at 37 °C, but the cost of this method is relatively high as the reagents of RPA and cas12a enzymes are still expensive. Aside from that, Bar-cas12a outperforms several traditional methods (e.g. PCR or HRM) for onsite testing. Additionally, it is also independent of expensive instruments and offer high accuracy, sensitivity, and robustness for single species authentication. Recently, CRISPR-cas technology-based nucleic detection also enabled multiplex detection for multiple viral detection for Dengue or Zika virus^31^. Multiplex detection is an elusive goal for molecular detection including species identification with multiple species in a single reaction.

Aside from being a highly specific, sensitive, rapid, and simple diagnostic tool, this approach has the inherent limitation of requiring an amplification process to increase a large amount of DNA target to activate the collateral activity of cas12a. Given that direct DNA extracts can be used as template to be performed by cas12a without DNA amplification, we strongly believe that the approach would be near the ideal method for rapidity. Hence, we purpose a concept to reduce DNA amplification step by using multiple gRNAs which can bind to different but specific DNA target, contributing to increasing amount of activated cas12a in the reaction.

In summary, our findings demonstrated that Bar-cas12a serve as immensely promising tool with highly specificity, sensitivity, speed, and simplicity for species discrimination/authentication in plant species especially in genus *Phyllanthus*. We proposed that this approach is a new shed of light in accommodating species discrimination/authentication for onsite testing which make us identify or distinguish plant species/commercial product in fields without the sophisticated equipment in two hours which is superior to several traditional methods.

## Methods

### Specimens and DNA extraction

*Phyllanthus* species including *P. amarus*, *P. urinaria*, *P. debilis* and *P. virgatus* were collected around Naresuan University, Phitsanulok, Thailand. These species were identified through a key from Flora of Thailand *Euphorbiaceae* (http://www.nationaalherbarium.nl/ThaiEuph/ThPspecies/ThPhyllanthusT.htm). The experiment and plant samples collection complied with guidelines of Department of Biology, Faculty of Sciences, Naresuan University. Their herbarium specimens were kept at the PNU plant herbarium, Department of Biology, Faculty of Science, Naresuan University (Table 1). In this study, *P. amarus* was used as a plant model to authenticate by the cas12a assay because it has been extensively used for medical purposes. Leaves were used for DNA extraction by Genomic DNA isolation kit (PureDireX, Taiwan). The quality and quantity of DNA obtained were measured by Nanodrop (Thermo Scientific, USA) and 1% agarose gel electrophoresis. DNA samples were diluted as 20 ng/ul and stored at − 20 °C for further use.

**Table 1:** Main morphological features and herbarium vouchers of *Phyllanthus* used in this study.

Species	Main morphological features	Herbarium voucher
*P. amarus*	Five sepals; capsule glabrous; leaf apical rounded	PNU-05739
*P. urinaria*	Six sepals; capsule scurfy-tuberculate; leaf apical obtuse	PNU-05740
*P. debilis*	Six sepals; capsule glabrous; leaf apical rounded	PNU-05738
*P. virgatus*	Six sepals; capsule glabrous leaf base obtuse or rounded	PNU-05741

### Design and synthesis of guide RNA for cas12a

To generate suitable gRNAs for cas12a assay, *trnL* region of the four species was conducted for multiple alignment by MultAlin (http://multalin.toulouse.inra.fr/multalin/)^32^. There were two significant points as guideline for gRNA design for species differentiation: (1) searching for protospacer adjacent motif (TTTV (V = A, G or C)) and (2) the sequences for DNA targets having variation among four species in the seed sequences (1–5 first bases next to PAM)^25^, given as gRNA-A and gRNA-B for specific *P. amarus* (Fig. 1A). The synthesis of gRNA was done by in vitro transcription (IVT) under double stranded (ds) DNA as a template. The dsDNA was constructed and synthesized from Integrated DNA technologies (IDT, USA) which consisted of three parts as (1) T7 promoter regions, (2) tracrRNA to incorporate with cas12a to form binary complex and (3) crRNA to bind with DNA target, forming a tertiary complex. These dsDNAs were used as template for RNA synthesis via in vitro transcription (IVT) by HiScribe T7 Quick (#E2050S, NEB, US). The synthetic gRNAs were purified to remove the impurities by the Monarch RNA Cleanup Kit (50 µg) (NEB, US). The synthetic gRNA products were measured for amount and purity by Nanodrop and 2% agarose gel electrophoresis and then adjusted for concentration to 10 µM for further study.

### In vitro cas12a assay

In this experiment, the concentration ratio of cas12a or cpf1 (#M0653T, NEB, US) and gRNA were varied to find out the suitable condition of in vitro digestion of cas12a. The ratio of cas12a and gRNA was constantly done at 1: 1 but the final concentrations were varied from 12.5 nM: 12.5 nM to 100 nM: 100 nM. Firstly, the binary complex between cas12a (cpf1) and gRNA was formed under admixture of 1X 2.1 NEB buffer, 100 nM cpf1, 100 nM gRNA-A or -B and then incubated at 37 °C for 10 min. Subsequently, 1 µl of 50 µM single stranded DNA reporter (ssDNA reporter) (FAM/TTATT/3IABkFQ) (IDT, USA) and 5 µl of DNA targets (RPA products) were added. Finally, the nuclease-free water was added to 24 µl and incubated at 37 °C for an hour. The cleavage of ssDNA reporter was determined under LED transilluminator to visualize the florescence signal by visible eye.

### Specificity and sensitivity determination of Bar-cas12a assay

To assess the specificity of cas12a assay for species authentication of *P. amarus*, gRNA-A and gRNA-B were compared by RPA products which were amplified from different *Phyllanthus* species DNA including *P. amarus*, *P. urinaria*, *P. debilis* and *P. virgatus*. TwistAmp Liquid Basic kit (TwistDx, England) was carried out to set a RPA reaction of 25-µl volume consisting of 1X reaction buffer, 1X probe E-mix, 1.8 mM, 0.48 µM *trnL*_RPAF, a forward primer (5′ TGTTGTCAATATTGACATGTAGAATGGGACTCTAT 3′), 0.48 µM *trnL*-RPAR, a reverse primer (5′ GCAGAGACTCAATGGAAGCTGTTCTAACAAACGG 3′), 1X core reaction mix, 20 ng of DNA template or plant extract and 14 mM magnesium acetate on the lid of PCR tube and added nuclease free water to 25 µl. The reaction was performed under heating box at 37 °C for 40 min. The RPA products were checked by 1.5% agarose gel electrophoresis, yielding approximately a 364–400 bp amplicon size (Figure S6). Afterward, amplified RPA products of different species were used as DNA targets for assessing the specificity of Bar-cas12 assay using gRNA-A or gRNA-B.

To evaluate the sensitivity of Bar-cas12a assay using gRNA-A and gRNA-B, initial concentration at 80 ng/µl of *P. amarus* DNA was done for ten-fold dilution, given final amount in range of 0.8 fg to 80 ng to amplify by RPA. Subsequently, RPA products were detected for *P. amarus* by Bar-cas12a. The fluorescence which indicates the presence of DNA target was recorded every minute for 2 h of incubation under realtime PCR and after completing the reaction of cas12a assay, the tubes was determined under LED transilluminator to visualize the florescence signal by visible eyes.

### Species authentication in admixture by Bar-cas12a

To test the species authentication performance of Bar-cas12a assay using gRNA-A and gRNA-B, the admixture between two species of *P. amarus* and *P. urinaria* was done in different amount percentage proportion, given as 100%:0%, 50%:50%, 25%:75%, 10%:90%, 2%:98% and 0%:100%, respectively. The initial DNA concentration of each species used to create an admixture was 10 ng/µl. Subsequently, these admixtures were employed to authenticate *P. amarus* by the Bar-cas12a using gRNA-A and gRNA-B with realtime PCR to acquire the fluorescence signal over time, and then PCR tubes were observed under LED transilluminator.

### Accuracy test of Bar-cas12a for species authentication

To evaluate the accuracy degree of Bar-cas12a using *trnL* to authenticate species, the different *Phyllanthus* species included *P. amarus* (n = 7), *P. urinaria* (n = 2), *P. debilis* (n = 2), *P. virgatus* (n = 1) and other species (*P. airy-shawii* (n = 2)*, P. acidus* (n = 1)*, P. emblica* (n = 2)*, P. reticulatus* (n = 2), *Phyllanthus* sp. (n = 1)) were used for DNA isolation, and these extracted DNA samples were blinded to identify *P. amarus* through Bar-cas12a using *trnL* as previously earlier. A person conducting the test did not know the actual species (DNA samples) and species prediction was based on the test results from Bar-cas12a using *trnL* assay. Obtained RPA products were visualized under 1.5% agarose gel electrophoresis stained by EtBr and then used to determine species of *P. amarus* via cas12a cleavage using an LED transilluminator and a realtime PCR machine. The accuracy and precision degree were calculated as follows.

$${\text{Accuracy\;rate}} = \frac{{{\text{Correctly\;predicted\;samples}}}}{{{\text{Total\;samples}}}} \times 100$$

$${\text{Precision\;rate}} = \frac{{{\text{The\;count\;of\;actual}}\;{\it P.amarus}\;{\text{which\;is\;correctly\;predicted\;as}}\;{\it P.amarus} }}{{{\text{Total\;samples\;which\;are\;predicted\;as}}\;{\it P.amarus}}} \times 100$$

## Supplementary Information

Supplementary Information.
