# Supplementary material for: Bar-cas12a, a novel and rapid method for plant species authentication in case of Phyllanthus amarus Schumach. & Thonn
Source: Sci Rep. 2021 Oct 22;11:20888. doi: 10.1038/s41598-021-00006-1 (PMC8536675; doi:10.1038/s41598-021-00006-1)
Supplement: Supplementary file 1 — Supplementary Information. [file 41598_2021_6_MOESM1_ESM.docx]

**Supplementary information file**

**Bar-cas12a, a novel and rapid method for plant species authentication: a case of *Phyllanthus amarus***

Kittisak Buddhachat^1,2,*^, Suphaporn Paenkaew^1^, Nattaporn Sripairoj^1^, Yash Munnalal Gupta^1^, Waranee Pradit^3^ and Siriwadee Chomdej^3,*^

^1^Department of Biology, Faculty of Science, Naresuan University, Phitsanulok, 65000, Thailand

^2^Center of Excellence in Research for Agricultural Biotechnology, Department of Agricultural Science, Faculty of Agriculture, Natural Resources and Environment, Naresuan University, Phitsanulok, 65000, Thailand

^3^Research Center in Bioresources for Agriculture, Industry and Medicine, Department of Biology, Faculty of Science, Chiang Mai University, Chiang Mai 50200 Thailand

*Corresponding author

Kittisak Buddhachat, Ph.D

Department of Biology, Faculty of Science, Naresuan University, Phitsanulok, Thailand

E-mail : [kittisakbu@nu.ac.th](mailto:kittisakbu@nu.ac.th)

Siriwadee Chomdej, Ph.D

Department of Biology, Faculty of Science, Chiang Mai University, Chiang Mai, 50200, Thailand

E-mail : siriwadee@yahoo.com

**Figure S1** **The synthesis of gRNA-A and gRNA-B by *in vitro* transcription with T7 RNA polymerase.**

The synthesized gRNAs were detected by 1.5% agarose gel electrophoresis using 1X TAE buffer at 120 V for 30 min. The products were stained by EtBr and visualized under a UV transilluminator.

**Figure S2** **DNA amplification of various *Phyllanthus* species by RPA on *trnL* loci.**

DNA’s four species of *Phyllanthus* were used for DNA amplification by RPA. The RPA products were separated by 1.5% agarose gel electrophoresis using 1X TAE buffer at 120 V for 30 min and RPA products were stained by EtBr and visualized under a UV transilluminator. Obtained RPA products were used as DNA targets for further evaluating the specificity of Bar-cas12a assay to authenticate *P. amarus*. *Phyllanthus amarus* (PA)*, Phyllanthus urinaria* (PU)*, Phyllanthus debilis* (PD)*, Phyllanthus virgatus* (PV)

**Figure S3** **DNA amplification of *Phyllanthus* *amarus* in different concentrations by RPA on *trnL* loci.**

DNA’s of *P. amarus* was diluted from 80 ng to 8 ag for DNA amplification by RPA. The RPA products separated by 1.5% agarose gel electrophoresis using 1X TAE buffer at 120 V for 30 min and RPA products were stained by EtBr and visualized under a UV transilluminator. Obtained RNA products were used as DNA targets for further evaluating the sensitivity of Bar-cas12a assay to authenticate *P. amarus*.

**Figure S4 DNA amplification of *Phyllanthus* *amarus* in *Phyllanthus urinaria*-contaminated samples by RPA on *trnL* loci.**

Different dilution ratios of *P. amarus* (PA) to *P. urinaria* (PU) were obtained in various percentage of contamination ranging from 100%:0% – 0%:100% of PA:PU. These admixtures were used as templates for DNA amplification by RPA. The RPA products were separated by 1.5% agarose gel electrophoresis using 1X TAE buffer at 120 V for 30 min and RPA products were stained by EtBr and visualized under a UV transilluminator. These RPA products were used for further validating species authentication of *P. amarus* in samples adulterated with *P. urinaria*.

**Figure S5. DNA amplification by RPA on agarose gel of the different *Phyllanthus* species to evaluate the accuracy of Bar-cas12a using *trnL*.**

The different *Phyllanthus* species were used for RPA amplification including *P. amarus* (PA), *P. urinaria* (PU), *P. debilis* (PD), *P. virgatus* (PV)*, P. airy-shawii* (PS)*, P. acidus* (PAc)*, P. emblica* (PE)*, P. reticulatus* (PR), *Phyllanthus* sp. (Psp). The RPA products were separated by 1.5% agarose gel electrophoresis using 1X TAE buffer at 120 V for 30 min and RPA products were stained by EtBr and visualized under a UV transilluminator. These RPA products were used for further species authentication of *P. amarus* by cas12a assay to find out the RPA product amplified from intact *P. amarus*.

Figure 6S. The primer binding sites for RPA amplification and DNA target for *P. amarus*-specific gRNA-A and gRNA-B.

The *trnL* sequences retrieved from GenBank of different five *Phyllanthus* species including *P. amarus* (PA), *P. urinaria* (PU), *P. debilis* (PD), *P. virgatus* (PV) and *P. reticulatus* (PR) were done for multiple alignment by MultAlin (<http://multalin.toulouse.inra.fr/multalin/>) to design RPA primer, given as trnL_RPAF and trnL_RPAR as well as *P. amarus*-specific gRNA under PAM site (5’TTTV3’). Red present the highly conserved region, blue indicate the partial conserved region, black is a base different from the conserved base at that position and dash (-) is the gap.
